# Association of genetic variants with patient reported quality of life and pain experience in patients in the UK NCRI Myeloma X Relapse [Intensive]) trial; an exploratory study

**DOI:** 10.1038/s41409-022-01738-y

**Published:** 2022-06-29

**Authors:** John A. Snowden, Sam H. Ahmedzai, Angela Cox, David A. Cairns, A. John Ashcroft, Cathy Williams, Jamie D. Cavenagh, Anna Hockaday, Julia M. Brown, Ian W. Brock, Treen C. M. Morris, Gordon Cook, on behalf of the National Cancer Research Institute Haemato-oncology Clinical Studies Group

**Affiliations:** 1 Department of Haematology, Sheffield Teaching Hospitals NHS Foundation Trust, Sheffield, UK; 2 Department of Oncology and Metabolism, Sheffield Medical School, The University of Sheffield, Sheffield, UK; 3 Leeds Cancer Research UK Clinical Trials Unit, Leeds Institute of Clinical Trials Research, University of Leeds, Leeds, UK; 4 Leeds Cancer Centre, Leeds Teaching Hospitals, Leeds, UK; 5 Mid-Yorkshire Hospitals NHS Trust, Wakefield, UK; 6 Nottingham University Hospitals NHS Trust, Nottingham, UK; 7 Barts Hospital NHS Trust, London, UK; 8 College of Myeloma (UK), United Kingdom Myeloma Forum, London, UK

**Keywords:** Medical research, Genetics research

## Abstract

The Myeloma X trial provided a platform to explore genetics in relation to systematic assessment of patient-reported outcomes at key points during salvage treatment in multiple myeloma (MM) patients. Blood DNA was obtained in 191 subjects for single nucleotide polymorphism (SNP) genotyping. By univariable analysis, the non-coding rs2562456 SNP, upstream of LINC00664, was associated with several relevant pain and health-related quality-of-life (HRQoL) scores at 100 days after allocation to consolidation with autologous stem cell transplantation or weekly cyclophosphamide. Presence of the minor (C) allele was associated with lower pain interference (*p* = 0.014) and HRQoL pain (*p* = 0.003), and higher HRQoL global health status (*p* = 0.011) and physical functioning (*p* = 0.007). These effects were not modified by treatment arm and were no longer significant at 6 months. Following induction therapy, the rs13361160 SNP near the *CCT5* and *FAM173B* genes was associated with higher global health (*p* = 0.027) and physical functioning (*p* = 0.013). This exploratory study supports associations between subjective parameters in MM with SNPs previously identified in genome-wide association studies of pain. Conversely, SNPs in candidate genes involved in opioid and transporter pathways showed no effect. Further studies are warranted in well-defined cancer populations, and potentially assisted by whole genome sequencing with germline analysis in routine diagnostics in haematological cancers.

## Introduction

Multiple myeloma (MM) forms ~10% of haematological cancers and is largely incurable. However, therapeutic advances in the last two decades have led to major extension of survival. With improving survival, MM is being experienced by an increasing proportion of patients as a chronic illness, and therefore supportive and symptomatic care is a cornerstone of disease management, particularly in relation to pain control. There is a significant symptom burden at all stages of disease, including pain, fatigue and other elements which impact directly on health-related quality of life (HRQoL) [1–3].

Pain in myeloma arises not only from infiltration of the bone marrow by malignant plasma cells, bone osteolysis and fracture, but also from side effects of treatments, particularly chemotherapy-induced peripheral neuropathy, and may continue to be significant in patients with stable disease who are off active treatment [4]. In animal models and clinical studies of patients with bone-related malignancy, experience of pain and other symptoms are the result of the interactions between a) host/tumour factors such as cytokines operating locally within the bone where the plasma cells are multiplying [5, 6]; b) the acute and chronic effects of anti-myeloma therapy [4, 7]; c) long-term potentiation and sensitisation in the ascending pain pathways in the spinal cord and brain, balanced by descending modulatory controls [8]; d) pharmacokinetic and pharmacodynamic consequences of analgesic absorption and metabolism [9]; e) a range of other circulating pathophysiological factors, ranging from renal function, endocrine, dietary as well as cytokines and mediators of inflammation which can impact on physiological mechanisms of pain [4, 10]

Examples of genetic factors contributing to pain experience include the three main opioid receptors (mu, kappa and delta) whose genes contain inherited variants that occur relatively frequently in human populations [11–14]. Such genetic variation has been associated with gender differences in the response to experimentally induced pain [15–17], and higher requirements for mu agonist drugs such as morphine and higher quantities of potentially toxic metabolites of morphine [18, 19]. While little is known about the effects of their SNPs in humans, kappa and delta-opioid receptors are considered important mediators in chronic pain situations and patients carrying alleles showing abnormal expression or function may also demonstrate aberrant pain experience. Like many other drugs, the absorption of opioids from the gastrointestinal tract into blood and thence across the blood-brain barrier is regulated by transporter proteins, and the function of these and subsequent drug metabolism also have a genetic basis [20, 21].

Genome-wide association studies (GWAS) carried out to date have not included cancer-related pain, but GWAS of post-surgical and chronic widespread pain have generated some potentially relevant candidate gene regions [22, 23]. Previous genetic studies of cancer pain have usually been carried out in patients with mixed tumour types, often in the advanced stages of illness, when many other factors can aggravate pain experience and confound the measurement of pain response. However, there are currently no published studies of SNPs or other genetic determinants and their effects on pain and analgesic response in patients with MM. There is a need for studies to be done in more tightly controlled populations with well-circumscribed treatments.

The UK National Cancer Research Institute Myeloma X trial was a phase III randomised controlled trial which recruited relapsed myeloma patients from 2008 to 2012. Its aims were to determine the role of a second or ‘salvage’ autologous stem cell transplant (sASCT) as a novel consolidation therapy in patients at first relapse following prior high-dose chemotherapy and ASCT. Myeloma X provided definitive evidence of the efficacy of a sASCT in terms of significantly improved time to progression, progression-free survival and overall survival, compared to conventional non-transplant consolidation (NTC) [24, 25]. A secondary aim of Myeloma X was to evaluate the impact of sASCT as compared to NTC with weekly oral cyclophosphamide on PROs relating to HRQoL and pain [26]. This linked HRQoL study confirmed that patients randomised to sASCT experienced a comparative reduction in quality of life and greater impact of treatment side-effects lasting for 6 months and for increased pain up to 2 years, after which patients who had received sASCT reported better outcomes than those receiving conservative treatment.

Myeloma X provided the only comprehensive picture of HRQoL before and after sASCT in patients with relapsed MM and highlighted the need to improve symptom management in the peri-transplant period [26]. The trial also provided a source of biological material by which the genetic basis of pain and analgesic response could be investigated in a well-defined homogeneous population of myeloma patients in conjunction with standard prospective assessments of pain, HRQoL, analgesic dosage and other symptom variables.

## Materials and methods

### Regulatory and ethical approval

Collection of pain and HRQoL data was intrinsic to the Myeloma X trial and consent for the use of translational research samples in additional genetic studies was incorporated to cover this and other studies associated with the trial (Multicentre Research Ethics Committee, UK; REC number: 07/S0703/66). The Myeloma X trial is registered with ClinicalTrials.gov, number NCT00747877 and European Clinical Trials Database, number 2006–005890–24.

### Patients and assessments

Details of the Myeloma X trial population are summarised in previous publications [24–26]. Between 16 April 2008 and 19 November 2012, of 297 patients registered, 288 patients consented to the QoL sub-study and 174 patients were randomly assigned to receive sASCT (*n* = 89; 88 consenting to QoL studies) or NTC (*n* = 85; 83 consenting)). Baseline demographic and disease characteristics were well balanced between the treatment groups, except for a higher proportion of patients with International Staging System III in the transplant group.

A secondary aim of Myeloma X was to determine the impact of the treatment strategies on pain and HRQoL [26]. These were variables collected from the outset of the Myeloma X trial, thus allowing exploratory analysis of these variables in relation to SNP genotypes. With respect to pain, the key outcome measures for pain were the Brief Pain Inventory (short form) (BPI-SF) [27], a well-established, validated instrument to assess pain severity (and its impact on several aspects of daily living). Key outcome measures for HRQoL relevant to this paper were the EORTC-QLQ-C30 [28] and its physical functioning, pain and fatigue scales. Questions 29 and 30 in the EORTC-QLQ-C30 are combined to give scores from 0–100 for ‘global health-related quality of life’. These key parameters were used as dependent variables in the main genetic analyses, alongside BPI scores. In both arms of the Myeloma X trial pain and quality of life questionnaires were administered at registration, after completion of induction chemotherapy, pre-randomisation and at 100 days, 6 months and 12 months post-randomisation and annually thereafter. Although attempts had been made to collect data on opioid and other analgesic consumption, the dataset was not sufficiently complete and therefore these variables were not included in the analysis.

### Patient samples

Blood DNA was available for 191 subjects for genotyping. Table 1 provides a summary of those with both successful genotyping and the relevant pain and QoL scores at the four-time points. The Haematological Malignancy Diagnostic Service laboratory in Leeds collected and managed all the tissue and blood samples from Myeloma X trial in full compliance with GLP standards. DNA was extracted from frozen whole blood using the Flexigene kit (Qiagen).

**Table 1:** Summary of age and gender distributions of consenting patients for whom SNP genotype and BPI Pain/HRQoL scores were available at each time point.

**SNP genotype and pain QoL score available**		**Male**			**Female**			
BPI worst pain	**Time point**	**median age**	**age range**	***n***	**median age**	**age range**	***n***	**total** ***n***
	registration	59	38–71	105	60	42–73	47	152
	end PAD	59	38–71	81	59.5	42–73	32	113
	100 days	59.5	41–72	68	61	42–73	19	87
	6 months	60	41–72	67	62.5	42–73	20	87
BPI least pain	registration	60	38–71	105	60	42–73	47	152
	end PAD	59	38–71	80	59.5	42–73	32	112
	100 days	59	41–72	67	61	42–73	19	86
	6 months	60	41–72	67	63	54–73	19	86
BPI average pain	registration	59	38–71	102	60	42–73	47	149
	end PAD	59	38–71	80	59.5	42–73	32	112
	100 days	59	41–72	67	61	42–73	19	86
	6 months	60	41–72	67	62.5	42–73	20	87
Pain interference	registration	59	38–71	103	60	42–73	46	149
	end PAD	59	38–71	75	59	42–73	32	107
	100 days	60	41–72	66	60.5	42–73	18	84
	6 months	60	41–72	68	63	51–73	19	87
QoL global health status	registration	60	38–72	116	60	42–73	51	167
	end PAD	59	38–71	90	59	42–73	37	127
	100 days	60	41–72	73	61	42–73	19	92
	6 months	60	41–72	69	61	42–73	23	92
QoL Physical functioning	registration	60	38–72	115	60	42–73	53	168
	end PAD	59	38–72	89	59	42–73	37	126
	100 days	60	41–72	74	60.5	42–73	20	94
	6 months	60	41–72	70	61	42–73	23	93
QoL pain	registration	60	38–72	116	60	42–73	53	169
	end PAD	59	38–72	91	59	42–73	37	128
	100 days	60	41–72	74	60.5	42–73	20	94
	6 months	60	41–72	70	61	42–73	23	93
QoL fatigue	registration	60	38–72	116	60	42–73	53	169
	end PAD	59	38–72	89	59	42–73	37	126
	100 days	60	41–72	74	60.5	42–73	20	94
	6 months	60	41–72	70	61	42–73	23	93
All trial participants	**registration**	**61**	**38–74**	**208**	**62**	**42–75**	**89**	**297**
	**end PAD**	**59.5**	**38–73**	**134**	**61**	**42–75**	**61**	**195**
	**100 days**	**60.5**	**40–73**	**112**	**62**	**42–73**	**36**	**148**
	**6 months**	**60**	**40–73**	**107**	**62**	**42–73**	**36**	**143**

### Genotyping

SNPs were selected in candidate genes relating to pain pathways (notably opioid receptors OPRM1, OPRD, TRPV1) and genes relating to drug absorption and metabolism (ABCB1, COMT), based on previous reports of association with pain and QoL in the literature, including two SNPs identified in GWAS of pain in non-cancer populations (rs2562456 near LINC00664, rs13361160 near CCT5, FAM173B). The selected SNPs and corresponding assays used are listed in Table 2.

**Table 2:** Selected SNPs and corresponding assay information.

Gene	Variant	SNP	Assay id	Reference
OPRM1	c.118A>G; p.Asn40Asp	rs1799971	C___8950074_1_	Campa et al. [21] Reyes-Gibby et al. [33]
OPRM1	intronic G>A	rs9479757	C__25472011_10	Nielsen et al. [34]
OPRD1	intronic G>A	rs2236861	C__15955109_10	Crist and Clarke [35]
LINC00664	upstream C>T	rs2562456	C___8166644_20	Kim et al. [31]
TRPV1	c.1753A>G; p.Ile585Val	rs8065080	C__11679656_10	Kim et al. [29]
CCT5, FAM173B	intergenic T>C	rs13361160	C__32226363_10	Peters et al. [23]
ABCB1	c.3645T>C; p.Ile1215=	rs1045642	C__7586657_20	Beer et al. [36]
ABCB1	c.2887T>G; p.Ser963Ala c.2887T>A; p.Ser963Thr	rs2032582	C__11711720D_40, C__11711720C_30	Campa et al. [21]
COMT	c.472G>A; p.Val158Met	rs4680	C__25746809_50	Reyes-Gibby et al. [33]

Selected SNPs and corresponding assays in candidate genes relating to pain pathways (OPRM1, OPRD, TRPV1), genes relating to drug absorption and metabolism (ABCB1, COMT) and SNPs identified in relevant GWAS (rs2562456, rs13361160).

Genotyping was carried out for each consenting patient registered in Myeloma X and with blood DNA available using the Applied Biosystems Taqman platform (Thermo Fisher Scientific). The assay for rs9479757 gave poor allelic discrimination and these data were not included, but call rates for all other SNPs were >98%. Nineteen (~10%) of samples were duplicated on the assay plates for all SNPs, and there was 1 discrepancy in genotype calls amongst the duplicates (<0.1%).

### Hypotheses and statistical analysis

We hypothesised that inherited genetic variants of relevant pain receptor pathways previously reported to be associated with pain and quality of life outcomes are associated with BPI pain scores and HRQoL in patients with relapsed MM. To test this hypothesis, pain (worst, least, average pain and pain interference from BPI-SF) [27] and HRQoL variables (Global Health Status, Physical Functioning, Pain and Fatigue in QLQ-C30) [28] were used.

Exploratory univariable linear regression with SNP genotype as explanatory variable was carried out for eight SNPs and eight pain and quality of life variables at four time points; study registration, end PAD, 100 days post-randomisation and 6 months post randomisation. SNP genotypes were coded as 0, 1, 2 for common homozygous, heterozygous and rare homozygous genotypes respectively. Multivariable regression was carried out to control for treatment arm, with NTC as the reference group. Statistical analyses were carried out in Stata Version 12 and all statistical tests were two-sided. The eight SNPs represent independent tests, but many of the outcome variables are correlated with one another. A guideline Bonferroni-adjusted *p* value threshold to help identify effects of interest would be 0.05/8 = 0.006. All *p* values are two-sided and are shown unadjusted.

## Results

### Association between inherited variants and pain and quality of life scores

**Fig. 1:**
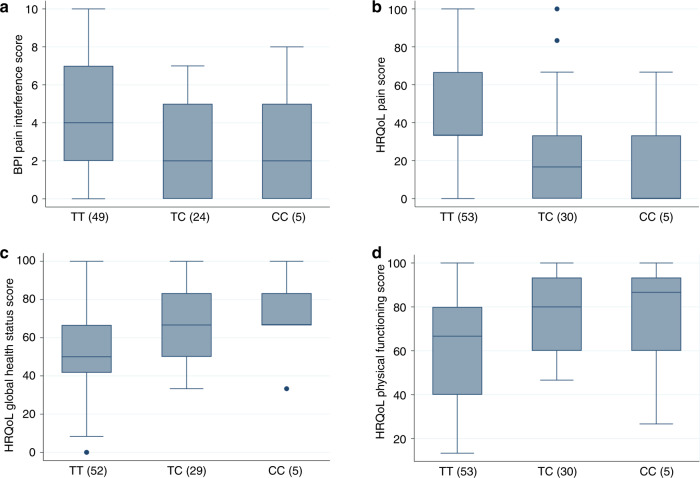
The SNP rs2562456 is associated with pain and quality of life scores at 100 days post-treatment allocation. Box and whisker plots by SNP genotype for **a** BPI pain interference score, **b** HRQoL pain score, **c** HRQoL global health status score, and **d** HRQoL physical functioning score, at 100 days. The *X*-axes show SNP genotype group and the number of individuals of each genotype in brackets. Regression *p* values for univariable analyses are as follows. **a**: 0.014; **b**: 0.011; **c**. 0.007; **d**: 0.003; see Table 3.

A summary of the age and gender distributions of consenting patients with SNP genotype and BPI Pain/HRQoL scores available is shown in Table 1. In the exploratory univariable analysis, there was suggestive evidence that the non-coding rs2562456 SNP upstream of LINC00664 on chromosome 19 was associated with a number of pain and HRQoL scores at 100 days after randomisation to sASCT or NTC (Fig. 1 and Table 3; full results in Supplementary Table 1). The presence of the minor C allele was associated with lower BPI pain interference score (*p* = 0.014) and HRQoL pain score (*p* = 0.003), and higher HRQoL global health status (*p* = 0.011) and physical functioning scores (*p* = 0.007). The graphs in Fig. 1 suggest a dominant genetic effect, in other words, carriage of one, or two, copies of the C allele have a similar effect on pain and HRQoL, although numbers of rare homozygous individuals are too small to formally test this hypothesis. No significant associations remained at 6 months (Supplementary Table 1). These results suggest that patients carrying at least one copy of the C allele of rs2562456 experienced less pain and better quality of life compared to those of the more common TT genotype at 100 days after randomisation, but that these genetic differences in outcomes disappeared by 6 months.

**Table 3:** Regression models for rs2562456 at 100 days post allocation and rs13361160 post-induction therapy.

100 days, rs2562456	Coefficient	Standard error	95% confidence interval	*p* value	Observations
BPI pain interference score					
effect of SNP	−1.4	−0.55	−2.5, −0.28	0.014	78
effect of treatment arm	1.04	0.51	0.04, 2.04	0.042	134
effect of SNP, controlling for treatment arm	−1.2	0.55	−2.3, −0.058	0.039	78
HRQoL global health status score					
effect of SNP	10.2	−3.9	2.4, 18.0	0.011	86
effect of treatment arm	−8.5	3.7	−16.0, −1.09	0.025	146
effect of SNP controlling for treatment arm	9.0	4.02	0.98, 17.0	0.028	86
HRQoL physical functioning score					
effect of SNP	11.0	4.0	3.1, 19.0	0.007	88
effect of treatment arm	−5.9	3.8	−13.0, 1.6	0.12	148
effect of SNP, controlling for treatment arm	10.06	4.1	1.9, 18.0	0.017	88
HRQoL pain score					
effect of SNP	−15.4	−5.0	−25.0, −5.5	0.003	88
effect of treatment arm	9.6	4.9	0.0, 19.0	0.05	148
effect of SNP, controlling for treatment arm	−15.0	5.2	−25.0, −4.3	0.006	88
**Post induction therapy, rs13361160**					
HRQoL global health status					
effect of SNP	5.83	−2.6	0.69, 11.0	0.027	127
HRQoL physical functioning					
effect of SNP	7.17	−2.83	1.56, 13.0	0.013	126

Results of univariable regression models for the effects of rs2562456 and treatment arm at 100 days, together with the SNP effects for the bivariable model including SNP and treatment. The reference group for the SNP effects is the common homozygous group and the reference group for treatment is the NCT with cyclophosphamide group. The results of univariable regression models for the effects of rs13361160 post-induction therapy are also shown.

We reported previously that global health status scores were better in the NTC group at 100 days post-randomisation [26]. We, therefore, explored whether the above rs2562456 SNP effects were modified by treatment arm. Table 3 therefore also shows the results of univariable regression models for treatment, together with the SNP effects where both SNP and treatment are included in the model. Based on these analyses, there is no evidence to suggest that the SNP effects are substantially modified by treatment.

**Fig. 2:**
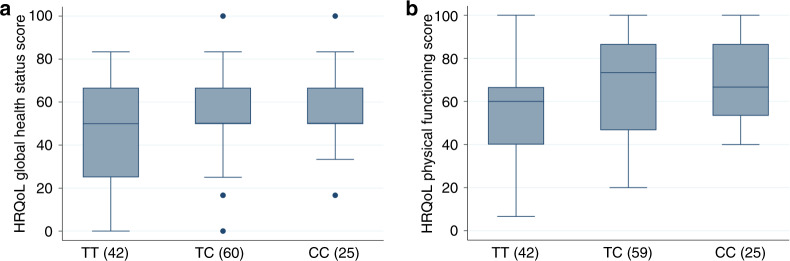
The SNP rs13361160 is associated with HRQoL global Health status and HRQoL physical functioning scores at the end of induction therapy. Box and whisker plots by SNP genotype for **a**: HRQoL global health status and **b**: HRQoL physical functioning post-induction therapy. The *X*-axes show SNP genotype group and the number of individuals of each genotype in brackets. Regression *p* values for univariable analyses are as follows. **a**: 0.027, **b**: 0.013; see Table 3.

We also identified some less significant SNP effects with individual pain and HRQoL scores at other time points (highlighted in Supplementary Table 1). Of these, only one SNP yielded nominally significant *p* values across more than one pain or HRQoL score. Following induction therapy, the minor C allele of the rs13361160 SNP near the *CCT5* and *FAM173B* genes was associated with higher global health (*p* = 0.027) and physical functioning scores (*p* = 0.013) at this time point (Fig. 2 and Table 3). Other nominally significant findings (*p* < 0.05) included the association of rs1799971 in OPRM1 with BPI Least Pain score and rs2562456 upstream of LINC00664 with HRQoL fatigue score, after induction therapy, and rs2035282 in ABCB1 with BPI Average Pain, and rs8065080 in TRPV1 with Pain Interference score, at registration (Supplementary Table 1).

## Discussion

The Myeloma X trial provided us a unique opportunity to study pain and HRQoL in a well-defined relatively homogenous cancer population all receiving a set of acute standardised anti-cancer interventions. A key difference between our study and previous ones in cancer and many other long-term conditions is that we have focused on a relatively earlier stage of disease and in the context of a well-defined clinical scenario, namely myeloma in first relapse undergoing consolidation treatment with sASCT or weekly cyclophosphamide.

Our exploratory study has provided evidence to support a role for some candidate SNPs identified in previous GWAS for pain, but, interestingly, we found only very minimal support for SNPs in candidate pain pathways such as opioid receptors. Because we also studied a range of HRQoL endpoints, we were able to identify gene associations beyond the pain outcomes that are normally reported.

Our previous results from the Myeloma X trial showed that at 100 days after randomisation global health scores were better in the NTC arm compared to the sASCT arm [26]. Interestingly, the more convincing SNP associations with pain and HRQoL were also observed at this time point. We found the minor (C) allele of the non-coding rs2562456 SNP upstream of the long non-coding RNA LINC00664 was associated with lower pain scores and higher global health status and physical functioning scores at 100 days, and there was no significant evidence of any difference between treatment arms, although power to test this is low. Somewhat consistent with this result, this SNP was previously identified in a small GWAS examining acute post-operative pain following dental surgery, which found that subjects who were homozygous for the minor allele of rs2562456 experienced a longer time before they required post-operative analgesia [29]. Further work needs to be done to understand the potential role of this SNP in cancer populations.

A SNP in rs13361160 SNP near the *CCT5* and *FAM173B* genes has previously been shown to be important in GWAS study of patients who had chronic widespread pain. We have now also identified this as being potentially relevant in MM patients, although it was associated not with pain scores, but rather with higher global health and physical functioning scores.

The results for both SNPs need to be interpreted with caution due to the large number of comparisons carried out in this exploratory study, and further research specifically in myeloma cohorts is required, in order to validate the present findings. The potential mechanisms of action are not clear, as non-coding SNPs may act by altering the expression of genes that are distant from the SNP in the genome. Thus, if the findings are validated, the next steps will be to identify the target genes through functional genomics, and to tease out the precise genetic models and biological mechanisms.

In contrast to the above results for two SNPs identified through GWAS, many of the positive gene associations with pain that have previously been reported in cancer patients were negative in this study. This may result from a lack of statistical power in the present study, as others have been larger. Alternatively, it may reflect the much more defined population and very specific treatments in the present study, compared to previous studies which have been in more heterogeneous settings. The Myeloma X trial did not standardise the opioid regimens for pain management which could explain why we failed to identify associations between pain outcomes and specific opioid receptor pathways. However, it should be noted that previously published studies exploring genetic influences on pain have mostly used heterogeneous samples of non-cancer populations, and in cancer, mixed primary sites and patients at different stages of disease and on differing anti-cancer or analgesic treatments [18]. Not surprisingly, therefore, the results of individual studies have been mixed and most later systematic reviews have concluded that only a few salient genetic signals stood out consistently [30, 31]. These include SNPs in genes associated with the pain pathways (notably opioid receptors) and genes relating to drug absorption and metabolism [32, 33]. It should be noted that there have been relatively few GWAS studies of pain to date, and none so far have been conducted in the cancer setting.

This study is subject to several limitations including an open-label design where lack of blinding may have relevance in relation to subjectively reported endpoints, using paper-based questionnaires subject to adherence, especially when patients are experiencing periods of increased illness and attrition rates as a possible source of bias. The sample size for this study was not specified a priori, and therefore results should be interpreted with caution. We have previously reported that randomisation to sASCT led to a significantly worse short-term HRQoL and pain outcomes compared to standard treatment; in addition, those patients who reported more acute treatment-related side effects experienced different HRQoL outcomes [26]. It is possible that by pooling both treatment arms for the current analysis, some potential signals may have been lost. However, compared to the full dataset of our earlier publication, the sample size for this study was judged to be too small for such subgroup comparisons. As stated above, it was not feasible to standardise the analgesic regimens for all the Myeloma X trial sites, and although attempts had been made to collect data on significant pharmacological parameters, such as opiates and other analgesia, the dataset was not sufficiently complete and therefore these variables were not accounted for and may have had an overriding or confounding influence.

In conclusion, although exploratory, with uncertain generalisability and reproducibility at present, our study paves the way for further research of HRQoL, pain and other symptomatology both in MM and potentially other cancers associated with pain. The ultimate benefit of this and subsequent research will be to identify patients who are more at risk of developing pain and other symptoms, or who are more likely to have variant responses to conventional medical analgesic interventions, in order to improve the patient experience as well as enhance survival. Further exploration is warranted to increase our understanding and provide therapeutic benefits in personalised care, both in myeloma-specific and in broader cancer settings. This may be assisted by whole genome sequencing with germline analysis in routine diagnostics in haematological and other cancers.

## Supplementary information

Supplementary table 1. Complete regression results organised by time point and by SNP with the results mentioned in the text highlighted.

## Data Availability

The datasets generated during and/or analysed during the current study are available from the authors on reasonable request (via corresponding author). No data were deposited for this specific exploratory study, Previous relevant Myeloma X clinical trial publications are fully referenced.
